# Antimicrobial resistance pattern, genetic distribution of ESBL genes, biofilm-forming potential, and virulence potential of *Pseudomonas aeruginosa* isolated from the burn patients in Tehran Hospitals, Iran

**DOI:** 10.11604/pamj.2020.36.233.21815

**Published:** 2020-07-30

**Authors:** Mohammad Shahbazzadeh, Elham Moazamian, Alireza Rafati, Masoud Fardin

**Affiliations:** 1 Department of Microbiology, Shiraz Branch, Islamic Azad University, Shiraz, Iran,; 2 Department of Microbiology, Ardabil Branch, Islamic Azad University, Ardabil, Iran

**Keywords:** *Pseudomonas aeruginosa*, antibiotic resistance, biofilm, ESBLs, virulence

## Abstract

**Introduction:**

according to the studies performed, researchers considered Pseudomonas aeruginosa (P. aeruginosa) as the major cause of infectious diseases like burn and wound infection that makes it one of the most threatening opportunistic pathogens. The present research aimed at investigating antimicrobial resistance, biofilm-forming abilities, and frequency of the genes contributed to bla_VEB-1_, bla_PER-1_, and bla_PSE-1_ genes and virulence of P. aeruginosa separated from the burn infections in Tehran, Iran.

**Methods:**

we evaluated the resistance of 156 P. aeruginosa isolates to fifteen antimicrobial agents and generation of the ESBL and MBL enzymes phenotypically based on the CLSI instructions. Moreover, the biofilm forming potential has been assayed in a microtitre plate. In addition, PCR has been used to examine the frequency of virulence-and biofilm-related genes. Furthermore, the PCR of bla_VEB-1_, bla_PSE-1_, and bla_PER-1_ genes has been amplified.

**Results:**

according to the results, 72.2% of P. aeruginosa isolates have been MDR and 35.6% and 55.5% have been positive for producing MBL and ESBL, respectively. Moreover, 67.8% have been positive for forming biofilms. It has been found that 15.3% isolates are ESBL-positive; from among them 60% belong to the females and 40% belong to the males. In addition, one and two isolates respectively harbored the bla_VEB-1_and bla_PER-1_genes.

**Conclusion:**

the present research outputs indicated the higher frequency of the multi drug resistance and higher percent of the virulence-related genes in the clinical P. aeruginosa isolates in Iran.

## Introduction

It is widely accepted that *Pseudomonas aeruginosa (P. aeruginosa)* is an opportunistic, Gram-negative, nonfermenting bacteria, which is a popular cause of infections in humans. In fact, *P. aeruginosa* leads to numerous infections like the infection of the urinary tract, respiratory system, and soft tissue, bacteraemia, dermatitis, and diverse systemic infections, in particular in the hospitalized patients and immuno-compromised people. Moreover, patients suffering from serious burns would have a specific susceptibility to this pathogen infection over the period of hospitalization, which frequently causes remarkable mortalities and morbidity [1, 2]. Some studies attributed greater death rate of *P. aeruginosa* infections to the bacterium ability to easy adaptation to the environmental condition, rapid development of the resistance to antimicrobials, as well as the production of diverse virulence agents [1, 3]. Other studies showed the higher genetic potent to the quick acquisition of the drug resistance and lower permeability of the cell walls of the pathogen to the anti-pseudomonal agents [4, 5]. Moreover, others believe that the multi-drug resistant (MDR) isolates of this bacterium may result in the life-compromising and, under a number of conditions, infections that could not be treated; thus, they are recently regarded one of the key problems in controlling infections [6-10].

However, while multiple beta lactamase enzymes have been obtained, *bla_PER-1_* and *bla_VEB-1_* are produced in lower frequency and gained clinical significance due to their resistance to the oxyimino betalactams [11]. In fact, the resistance to carbapenems would be very important due to the operations versus the Gram-positive and Gram-negative isolates. We note that as in the case of *Acinetobacter baumannii* and *Klebsiella pneumoniae*, different systems should be combined in *P. aeruginosa* in order to achieve greater resistance to carbapenems [12]. Moreover, *P. aeruginosa* possess very high cell-related and extra-cellular virulence parameters. For example, Exotoxin A that is one of the main virulence factors of *P. aeruginosa* encoded by *toxA* gene suppresses synthesizing the protein. On the other hand, exoenzyme S that is encoded by the *exoS* gene has been considered as one of the key virulence factors contributed to the burn infection. In fact, the above cytotoxin modifies cytoskeleton performance of the host cell and causes bacterial invasion, dissemination, and colonisation over the infections period [13]. Based on a study in the field, a zinc metallo-protease encoded by the *lasB* gene elastolytically activates on the host tissues. In addition, alkaline protease, which is encoded by *aprA* gene and phospholipases C that is encoded by *plcN* and *plcH* genes, make easy the bacterial disseminations [6].

Interestingly, *P. aeruginosa* generates diverse enzymes and exotoxins. Moreover, it possesses considerable abilities to form biofilm. Additionally, a variety of bio-molecules like proteins and poly-saccharides in the biofilm matrix would protect bacteria from the immune responses of the host and from anti-microbials. Furthermore, alginate, which is encoded by *algD* gene, is one of the popular kinds of polysaccharides and is possibly exist in the structures of the biofilm. Furthermore, a neutral-charge exopolysaccharide is encoded by *pslA* gene, which supports the structure over the initial phase of the biofilm generation and facilitate the cell to cell and the cell to substrate binding. As a result of such a condition, it is a hard task to heal infection associated with the bio-film forming strains because it may result in severe concerns in the burn hospitals [14, 15]. The present research aimed at detecting the genes which encode the class A extended spectrum beta-lactamases (ESBLs) of *bla_VEB-1_, bla_PER-1_* and *bla_PSE-1_*, as well as investigating the patterns of the antimicrobial medicine resistance and presence of some significant virulence agents amongst *P. aeruginosa* separated from the burn patients in Tehran Province, Iran.

## Methods

**Bacterial analyses:** our research involved 176 clinical samples of the isolates of *P. aeruginosa*, which have been collected between May 2017 and December 2018 from the patients suffering from the burn infection in Tehran hospitals, Iran. Therefore, standard microbiological procedure has been used to isolate each *P. aeruginosa* sample and recognize by API 20NE (BioMerieux: France). Then, they stock-piled in Luria-Bertani broth medium (Merck: Germany) consisting of 15% glycerol at-70°C.

**Antimicrobial susceptibility experimentation:** we used the common Clinical and Laboratory Standard Institute (CLSI) instructions to determine *P. aeruginosa* resistance to the popular antimicrobial factors [7, 16, 17]. Thus, we evaluated 12 antimicrobials like gentamicin, amikacin, cefotaxime, ceftazidime, cefoxitin, ceftriaxone, meropenem, imipenem, trimethoprim/ sulfamethoxazole (SXT), piperacillin, ciprofloxacin, and erythromycin (Mast, UK). In addition, we utilized the *P. aeruginosa* ATCC 27853 reference strain as the control. Moreover, double disk diffusion technique through the cefotaxime (30μg) and ceftazidime (30μg) disks themselves and combined with clavulanic acid (10μg) on a MuellerHinton agar have been used to detect the production rate of ESBL. Consequently, a positive test output has been determined as a ≥5mm enhancement in the zone of inhibition (ZOI) diameter in comparison to the disk with no clavulanic acid. Furthermore, the production of the metallo-β-lactamase (MBL) enzyme has been assayed using an imipenem-ethylene diamine tetraacetic acid (IPM-EDTA) double-disk synergy experiment. Finally, those isolates with the enhanced ZOI diameter with IPM-EDTA in comparison to the imipenem-only disks have been regarded as the MBL-producers [16, 18].

**Assaying the biofilm formation:** it is notable that the biofilm formation has been assayed in a microtitre plate. To sum up, we diluted the standard overnight culture (1.5x10^8^CFU/mL) 100-fold in the brain heart infusion broth. Then, through all culture dilutions, 200μL has been transported in the separate wells of a 96-well flat-bottomed polystyrene plate (Sigma-Aldrich) and incubation has been done at 37°C for 48h. After the dilution has been incubated, we removed planktonic bacteria, and used sterile physiological saline to mildly washing the wells 3 times. Then, methanol has been used to fix the accompanied organisms for 20 minutes. Later, all wells have been stained with the crystal violet (CV), rinsed, and then the biofilm-related CV has been de-stained in 1mL of ethanol: acetone (95: 5, v/v). Finally, we measured the optical density at 600nm (OD600) of the blended solution. In the next stage, the isolates with OD600 > 0.625 have been categorized as the biofilm producers [19].

**PCR amplification of the virulence-related genes:** it should be noted that PCR through the gene-specific primers have been exploited for evaluating the presence of virulence-related genes like *pslA* and *algD* (biofilm related genes), *exoS* and *toxA* (exotoxin encoding genes) and *lasB, aprA*, plcN, and plcH (encoding alkaline protease, phospholipase, and elastase C enzymes) [19-21]. Table 1 reports the sequences of the oligonucleotide primers employed in our research. In addition, the extracted nucleic acid has been utilized as the template DNA for PCR. Then, PCR has been administered in a total volume of 25 μL consisting of 5 μL of enzyme buffer (10x), 0.5 μL of dNTPs (10 mM), 2 μL of template DNA (2 μg), 3 μL of forward and reverse primers (10 pmol), 14 μL of deionized water, and 0.5 μL of enzyme (2.5 U). Of course, all genes have been amplified individually. Notably, thermal cycler program accounted for a primary denaturation at 95°C for five minutes, thirty cycles of denaturation at 95°C for 60 seconds, annealination at 55°C for 45 seconds, and extension at 72°C for 75 seconds accompanied by the last extension stage at 72°C for ten minutes. Finally, electrophoresis on a 1% agarose gel has been used to detect PCR products.

**Table 1:** the oligonucleotide primers used in the study

Gene	Primer Sequence (5' to 3')	Amplicon Size (bp)	Reference
*aprA*	F: GTCGACCAGGCGGCGGAGCAGATA R: GCCGAGGCCGCCGTAGAGGATGTC	993	[21]
*exoS*	F: CTTGAAGGGACTCGACAAGG R: TTCAGGTCCGCGTAGTGAAT	504	[20]
*lasB*	F: GGAATGAACGAGGCGTTCTC- R: GGTCCAGTAGTAGCGGTTGG	300	[20]
*plcH*	F: GAAGCCATGGGCTACTTCAA R: AGAGTGACGAGGAGCGGTAG	307	[20]
*plcN*	F: GTTATCGCAACCAGCCCTAC R: AGGTCGAACACCTGGAACAC	466	[20]
*pslA*	F: CACTGGACGTCTACTCC GACGATAT R: GTTTCTTGATCTTGTGCAGGGTGTC	1119	[19]
*toxA*	F: GGTAACCAGCTCAGCCACAT R: TGATGTCCAGGTCATGCTTC	325	[20]
*algD*	F: ATGCGAATCAGCATCTTTGGT R: CTACCAGCAGATGCCCTCGGC	1310	[20]
*bla_PER-1_*	F: ATGAATGTCATTATAAAAGCT R: TTAATTTGGGCTTAGGG	927	[10]
*bla_PSE-1_*	F: AATGGCAATCAGCGCTTC R: GCGCGACTGTGATGTATA	699	[10]
*bla_VEB-1_*	F: CGACTTCCATTTCCCGATGC R: GGACTCTGCAACAAATACGC	624	[10]

**Amplifying the PCR of the *bla_VEB-1_, bla_PER-1_* and *bla_PSE-1_* genes:** according to the research design, PCR has been carried out to amplify the *bla_VEB-1_, bla_PER-1_*, and *bla_PSE-1_* genes with certain primers [22]. Moreover, the reaction mix (PCR master mix) involved MgCl_2_ (50 mM) = 1.5 μL, 10xPCR buffer = 2.5 μL, forward and reverse primers (each with 100 μM) = 2.5 μL, dinucleotide triphosphate (dNTP) (10 Mm) = 0.75 μL, DNA template = 1 μL, Taq DNA polymerase (5 U/μL = 0.2 μL, and nuclease-free H2O = 14.05 μL.

**Statistical analyses:** we used χ^2^test to compare the correlation between the biofilm forming potent and distribution of the virulence genes as well as pattern of the medicine resistance pattern. For this reason, P<0.05 has been estimated as statistically significant.

## Results

According to the detection of antimicrobial resistance profile to twelve diverse antimicrobials, 112 (63.6%) of the *P. aeruginosa* isolates have been MDR. Of course, the tested isolates had the greatest resistance to Cefoxitin (161; 91.4%) and SXT (138; 78.4%) (Table 2). In addition, Piperacillin and Amikacin have been identified as the most potential antimicrobials versus the clinical isolates of *P. aeruginosa*. Moreover, the phenotypic assays respectively showed the positivity of 65 (36.9%) and 91 (51.7%) of isolates for producing MBL and ESBL production. It should be noted that from among 176 experimented isolates, 107 (60.8%) have been positive for forming the biofilm. It has been shown that each MDR isolate is the biofilm producer. Moreover, resistance to each experimented antimicrobial in the biofilm forming isolates has remarkably increased compared to the antimicrobials in the non-biofilm producing isolates. In addition, formation of biofilm has been observed in all (100%) gentamicin-, amikacin-, imipenem-, and piperacillin-resistant isolates.

**Table 2:** antimicrobial resistance properties of *P. aeruginosa* clinical isolates (n=176) and distribution of biofilm formation in resistant isolates

Antibiotics	Resistant isolates [n (%)]	Biofilm-producers [n (%a)]
Gentamicin	41 (23.3)	41 (100)
Ciprofloxacin	81 (46)	65 (80.2)
Amikacin	21 (11.9)	21 (100)
Erythromycin	76 (43.1)	63 (82.9)
Ceftazidime	121 (68.7)	113 (93.3)
Meropenem	69 (39.2)	65 (94.2)
Imipenem	77 (43.7)	77 (100)
Cefotaxime	99 (56.2)	85 (85.8)
Piperacillin	35 (19.8)	35 (100)
Ceftriaxone	79 (44.8)	71 (89.8)
Cefoxitin	161 (91.4)	125 (77.6)
SXT	138 (78.4)	121 (87.6)

SXT, trimethoprim/sulfamethoxazole. a: percentage of biofilm-producers among isolates resistant to that agent.

Results showed that each MBL-positive isolate and 85.2% of the phenotypically ESBL-positive isolates have been the biofilm producers. Table 2 gives distribution of the biofilm forming resistant isolates and anti-microbial resistance profiles of the *P. aeruginosa* isolates. Table 3 illustrates frequency of the virulence-related genes and distribution in the biofilm producing and non-biofilm producing *P. aeruginosa* isolates. In fact, virulence-and biofilm-related genes have been recognized in greater than 50% of the *P. aeruginosa* isolates so that *toxA, lasB*, and *plcH* had the greatest frequency. It should be noted that each virulence gene has been more common in the MDR and biofilm forming phenotypes. According to the results, from among the 91 ESBL-positive burn patients isolates, 29.6% of them (n = 27) harbored *bla_PER-1_* gene (925 bp) that observed in Tehran hospitals in Iran. In addition, amplification of 19.7% (n = 18) of the isolates has been seen in the *bla_VEB-1_* (634 bp); any of the ESBL producers did not amplify the *bla_PSE-1_* gene.

**Table 3:** distribution of the virulence- and biofilm-related genes in the biofilm producing and nonbiofilm producing *P. aeruginosa* isolates

Gene	Biofilm Formation n (%a)	
	Positive (107)	Negative (56)
algD (96)	96 (100)	0(0)
aprA (78)	56 (71.8)	22 (28.2)
exoS (108)	90 (83.3)	18 (16.6)
lasB (128)	98 (76.5)	30 (23.4)
plcH (116)	83 (71.5)	33 (28.4)
plcN (85)	50 (58.8)	35 (41.1)
pslA (92)	80 (86.9)	12 (13)
toxA (139)	96 (69)	43 (30.9)

a: percentage among isolates positive for that gene

## Discussion

It is widely accepted that antimicrobial resistance is a key issue in treating the infectious illnesses throughout the world. In fact, *P. aeruginosa* has an inherent resistance to several antimicrobials as a result of lower leakage of external membrane, fixed expression of numerous efflux pumps, and generation of diverse antimicrobial inactuating enzymes. Moreover, this pathogen possesses higher potency of producing biofilm, which results in laborious antimicrobial permeability and hard accessibility to bacteria. Therefore, we examined resistance to the antimicrobials, biofilm formation, the presence of ESBL and carbapenemase encoding genes, and frequency of diverse virulence related genes in the clinical isolates of *P. aeruginosa*. Outputs achieved in 90 isolates showed that 112 (63.6%) are MDR. In fact, piperacillin and amikacin have been antimicrobials with the highest potential. In addition, we found the increased resistance to meropenem, imipenem, and cephalosporins. However, some studies demonstrated the enhanced MDR *P. aeruginosa* strains and resistance to carbapenems, the selected antimicrobials to treat the *P. aeruginosa* infections throughout the world [1, 8, 23, 24]. Of course, other investigations published the great pervasiveness of cephalosporin-resistant *P. aeruginosa* because of the higher utilization of the β-lactam antimicrobials [4, 25]. Moreover, a 10-year retrospective study conducted on the *P. aeruginosa* isolates gathered from the burn patients, ceftazidime, and meropenem exhibited the highest rate of enhancement of resistance [26]. It is notable that the greater pervasiveness of the resistance to the β-lactams may influence clinical outcomes of the infections generated by such bacterium. However, recent investigations implied the correlation between the increased rate of death and the generation of ESBL in bacteria that results in the community-acquired infections like *P. aeruginosa* [27].

According to our phenotypic assays, 60.8% of the isolates could form biofilms. Moreover, frequencies of *algD* and *pslA* in the biofilm forming isolates respectively have been 100% and 86.9%. As shown, percent of the biofilm forming isolates correspond to the Emami *et al*. study who observed 70% biofilm-forming capacity in the burn isolates [20]; however, the *pslA* gene frequency increased in our research. Additionally, Ghadaksaz *et al*. found detection of the biofilm generation in 47.1% of the isolates; that is, detection of *pslA* and *algD* genes in 83.7% and 87.5% of the isolates [28]. This research illustrated that the experimented bacteria are positive for *exoS* (90, 83.3%), *toxA* (96, 69%), *plcH* (83, 71.5%), *lasB* (98, 76.5%), and *plcN* (50, 58.8%). Moreover, frequency of the virulence genes, which encode exoenzyme S, exotoxin A, phospholipase C, and elastase remarkably enhanced in the biofilm forming isolates in comparison to the nonbiofilm forming isolates. Therefore, the research suggested higher virulence, greater potency, and capability of the resistance of several antimicrobial agents of the biofilm forming strains (P < 0.05). Based on the outputs, the *toxA* gene frequency has been compatible with the frequency in the wound infections of the *P. aeruginosa* isolates published by Amirmozafari *et al*. Nikbin *et al*. and Mitov *et al*. [3, 29, 30]. In addition, *exoS* frequency has been consistent with the outputs published in the Mitov *et al*. study about the wound isolates of *P. aeruginosa* [3].

Nevertheless, frequency of *lasB* and the *plcH* genes decreased in our research. In contrast, *lasB* and *plcH* had considerably greater frequency than the isolates observed in the Ullah *et al*. study [31]. As stated by Fazeli and Momtaz, from among the 26 burn isolates of *P. aeruginosa*, 9 (34.6%) and 13 (50%) respectively have been positive for *plcN* and *plcH* [6] that is less than the ones reported in our research. Put differently, one of the studies conducted in Poland (2009) showed the positiveness of 100% of the wound infection from the *P. aeruginosa* isolates for *plcN* and *plcH* [32]. Results showed that nearly 50% of the isolates are resistant to the third-generation cephalosporins; however, just one *bla_VEB-1_* and two *bla_PER-1_* positive isolates could be identified, which demonstrates possible cooperation of the presence of other enzymes like ESBLs, AmpC, and metallo betalactamases or mechanisms such as the efflux pumps for resistance of cephalosporin to such an event. Nonetheless, ceftazidime resistance isolates were observed among 51 of Bacteria, of which 5 were ESBL producers. Of course, additional resistance mechanisms or enzymes possibly contribute to the above phenomenon. Moreover, the antibiotic susceptibility profile has been similar for 27 *bla_PER-1_* positive isolates, which indicates the incidence of the associated isolates. In addition, results revealed no amplification of the *bla_PSE-1_* gene by the ESBL producers [22].

## Conclusion

Based on the outputs obtained in the present research, an increased frequency of multi-drug resistance and the increased percent of the virulence-related genes have been observed in the clinical *P. aeruginosa* isolates in Iran.

### What is known about this topic

- P. aeruginosa is one of the most common causes of hospital-acquired infections and is commonly isolated bacteria in acute and chronic wound infections, respiratory infections, and medical device surfaces;
- P. aeruginosa produces diverse extracellular toxins and enzymes, and possesses abilities to form biofilm;
- Isolates of P. aeruginosa have acquired resistance to beta-lactam antibiotics.

### What this study adds

- 72.2%, 35.6% and 55.5% of P. aeruginosa were MDR, MBL and ESBL, respectively;
- The most prevalent ESβL-encoding gene was bla_PER-1_(29.6%).
